# Tumor-specific donor lymphocyte infusion therapy with allogeneic T cells utilizing novel retrovirus vector silencing endogenous TCR expression

**DOI:** 10.1186/2051-1426-2-S3-P20

**Published:** 2014-11-06

**Authors:** Hiroaki Ikeda, Hiroaki Ueno, Ayumi Kawamura, Naoko Imai, Sachiko Okamoto, Junichi Mineno, Kazutoh Takesako, Hiroshi Shiku

**Affiliations:** 1 Mie University Graduate School of medicine, Department of Immuno-Gene Therapy, Tsu, Japan; 2 Takara Bio Inc., Otsu, Japan; 3 Department of Medicine, Division of Hematology/Oncology, The Tisch Cancer Institute at Hess Center for Science and Medicine,Icahn School of Medicine at Mt. Sinai, Japan

## Background

Donor Lymphocyte infusion (DLI) is a therapy for the patients with relapsed hematological malignancy after allogeneic hematopoetic stem cell transplantation. However, the development of Graft-Versus-Host Disease (GVHD) is a serious adverse event and the efficacy is limited when one needs to control the GVHD. To inhibit the development of GVHD with increased tumor-specificity of transferred allogeneic lymphocytes, here we demonstrate the development of DLI with lymphocytes engineered to express tumor-specific T cell receptor (TCR) in combination with decreased GVHD-inducing potential utilizing the novel retrovirus vector (siTCR vector) that specifically silences endogenous TCR in gene-engineered T cells.

## Methods

Human PBMC were transduced with a high affinity TCR specific to a cancer/testis antigen, NY-ESO-1, by the retrovirus vector with siRNA specific to the endogenous TCR. Resulting TCR gene-transduced T cells were examined for their reactivity to allogeneic LCL by ^3^H uptake proliferation assay. Immunodeficient NOG mice were inoculated with a NY-ESO-1-expressing human melanoma cell line NW-MEL-38, received TCR gene-transduced T cells, and monitored for tumor growth and the development of GVHD.

## Results

Human lymphocytes that were genetically engineered to express a high affinity NY-ESO-1-specific TCR with siTCR vector showed reduced expression of endogenous TCR associated with the dramatically diminished reactivity to allogeneic lymphocytes. When administrated into NOG mice, these TCR gene-transduced T cells induced tumor regression without the development of GVHD.

## Conclusions

The results here suggest that the allogeneic T cells transduced with a tumor-specific TCR by siTCR vector showed diminished GVHD potential. These T cells will be applicable to the donor lymphocytes infusion therapy after allogeneic stem cell transplantation for the treatment of hematological malignancy, providing diminished GVHD and increased tumor eradication.

